# Patterns, Potential Teratogenicity, and Associated Factors of Drugs Prescribed to Pregnant Women Attending Antenatal Care Units in Debre Tabor Comprehensive Specialized Hospital, Debre Tabor, Northwest Ethiopia

**DOI:** 10.1155/2024/5577862

**Published:** 2024-11-11

**Authors:** Muluken Adela Alemu, Woretaw Sisay Zewdu, Yared Andargie Ferede, Mulugeta Molla Zeleke, Teklie Mengie Ayele, Abraham Nigussie Assefa, Tirsit Ketsela Zeleke, Achenef Bogale Kassie

**Affiliations:** ^1^Department of Pharmacy, College of Health Sciences, Debre Tabor University, PO Box 272, Debre Tabor, Ethiopia; ^2^Department of Pharmacy, College of Health Sciences, Debre Markos University, PO Box 269, Debre Markos, Ethiopia

**Keywords:** Debre Tabor Comprehensive Specialized Hospital, pregnancy, teratogenicity

## Abstract

**Background:** About 80% of pregnant women use at least one medication during their pregnancy period. Many drugs that are not allowed to be used during pregnancy (from FDA Pregnancy Categories D and X) were used. Irrational use of these drugs during pregnancy may result in different birth defects, as explained by thalidomide and diethylstilbestrol's tragedy. Knowledge of drug utilization and associated factors that affect the pattern is important to enhance rational prescribing. But information about prescription patterns and associated factors among pregnant women is scarce in the Debre Tabor area and generally in Ethiopia.

**Objective:** This study was aimed at assessing drug prescription patterns, potential teratogenicity, and associated factors among pregnant women attending the antenatal care unit at Debre Tabor Comprehensive Specialized Hospital, Debre Tabor, Northwest Ethiopia.

**Methods:** A retrospective cross-sectional study design was performed on 359 pregnant women attending antenatal care units from June 01, 2022, to August 30, 2022, in the hospital. Necessary data were obtained through a questionnaire by reviewing the medical charts of the women. Analysis of the data was performed using SPSS Version 23. The association of the independent variables to medication use was assessed using multivariate logistic regression. A *p* value of less than 0.05 was considered significant.

**Results:** Most of the study participants (325/359) were married (90.5%). From a total of 359 participants, 350 (97.5%) were prescribed with drugs. About 64% (385/602) of the prescribed medications were iron and vitamins. The most commonly prescribed medications are iron and folic acid combination (340/602, 56.5%), albendazole (48/602, 8%), mebendazole (37/602, 6.1%), omeprazole (33/602, 5.5%), followed by amoxicillin (32/602, 5.3%). The majority (79.3%) of the drugs were from FDA Pregnancy Categories A and B. Prescribed drug utilization was more probable in women who first visited the facility at their second (AOR = 2.91, 95% CI [1.12–6.64]) and third trimesters (AOR = 4.32, 95% CI [1.37–6.81]), had chronic illness (AOR = 7.54, 95% CI [2.34–14.68]), and live in rural areas (AOR = 2.47, 95% CI [1.56–8.43]).

**Conclusion:** The study revealed that the prescription pattern in the hospital is in line with the WHO reference. Age, gravidity, number of ANC visits, first visit to the facility, presence of chronic illness, educational status, and residency were significantly associated with prescription drug use in pregnant mothers. But still, some pregnant women received drugs that may have teratogenicity risk (FDA Category C).

## 1. Introduction

### 1.1. Background

Teratogenesis is a birth defect characterized by an abnormal structure or function of the body/body system in the developing embryo or fetus [1]. It may be manifested as growth retardation, transplacental carcinogenesis, and death of the embryo. A teratogen is any agent that may cause a birth defect. Many agents may cause teratogenesis, like chemical agents, including drugs, infections, in utero damage, and maternal disorders. The use of drugs in pregnancy is a growing concern due to the increasing risk of teratogenicity [2, 3].

A varying number of pregnant women use prescription drugs in different health settings. It is estimated that about 44%–99% of pregnant women use at least one medication during their pregnancy period. But the safety of these drugs is in question. A study found that many pregnant women took analgesics and antibiotics, of which a significant number is from Categories D and X [4, 5]. As a part of history, thalidomide, which was used for the treatment of anxiety and insomnia and as an antiemetic, was associated with a congenital anomaly, phocomelia, in thousands of children exposed to the uterus [6].

To ensure appropriate use of drugs during pregnancy, the Food and Drug Administration (FDA) categorized drugs into different categories (A, B, C, D, and X). Category A revealed no risks in adequate and well-controlled human studies on pregnant women, and Category B implies animal reproduction studies have shown no risk to the fetus as far as no adequate studies are available on pregnant women. On the other hand, Category C implies an adverse effect on the animal fetus but no adequate and well-controlled studies in humans. Medicines in Category D have evidence of fetal risk in pregnant women, but potential benefits may warrant the use of a drug in pregnant women despite potential risks, and those in Category X are known to have evidenced fetal risk [4, 7]. Birth defects are caused by several causes, of which 1% is due to irrational use of drugs [7, 8]. Irrational use of drugs during pregnancy may cause termination of pregnancy, birth defects, and defects that may appear later in life [9]. Rational use is to mean the right dose for the right indication at a reasonable price.

Many pregnant women use drugs during their pregnancy [10]. Studies conducted in Ethiopia at Tigray, Bahir Dar, and Addis Ababa showed 71.3%, 87.5%, and 88.4% of pregnant women use at least one drug during pregnancy, of which about 4%, 0.5%, and 11% are from Categories D or X, respectively [11–14]. Tocolytic agents, analgesics, and drugs for chronic diseases are the most prescribed drugs next to vitamins and iron for pregnant women [13]. Different studies show that several factors are associated with the pattern and prescription of drugs to pregnant women, and among these are multigravida, multiple visits to health facilities, health facility visits in the second trimester, women with comorbidities, and women with the age range 30–42 [14].

Knowledge of drug utilization and associated factors that affect the pattern is important to enhance rational prescribing for prescribers and pregnant women, which in turn reduces the harm to the fetus due to irrational utilization of the drugs. Such studies are conducted in some regions of Ethiopia, including Mettu, Adigrat, and Addis Ababa, but not in Debre Tabor. Therefore, this study was conducted to evaluate drug utilization patterns and associated factors among pregnant women in the Debre Tabor area, Ethiopia.

## 2. Materials and Methods

### 2.1. Study Design, Period, and Study Setting

A retrospective cross-sectional study design was used in reviewing the charts of pregnant women attending antenatal care (ANC) units either to begin or continue follow-up on June 01, 2022, to August 30, 2022, in Debre Tabor Comprehensive Specialized Hospital, Debre Tabor, Ethiopia. The hospital is located in Debre Tabor town, South Gondar zone, Amhara regional state, Northwest Ethiopia, 667 km far from Addis Ababa and 102 km from Bahir Dar, the capital of Amhara regional state. It gives services to about 2.5 million people per year. The hospital also serves as a teaching center for students of Debre Tabor University.

### 2.2. Source Population

The source population included all medical records of pregnant women who have visited ANC units of DTCSH from June 01, 2022, to August 30, 2022.

### 2.3. Study Population

The study population was the selected medical records having complete information on pregnant women who came to ANC units of DTCSH from June 01, 2022, to August 30, 2022.

### 2.4. Inclusion and Exclusion Criteria

All complete medical records of pregnant women who attended ANC units of DTCSH from June 01, 2022, to August 30, 2022, were included in the study. On the other hand, records of pregnant women who had incomplete information were excluded from the study.

### 2.5. Sample Size Determination and Sampling Procedures

The sample size was computed using a general formula for a single population proportion (*n* = *Z*^2^*pq*/*d*^2^) considering 50% prevalence since there is no benchmark study. The sample size was 384, assuming a 5% margin of error at a 95% confidence interval.

Where p = the proportion of drug use among pregnant women =0.5, q = (1-p), Z = confidence interval =1.96, d = margin of error =0.05, n = sample size.

$$\begin{array}{cc} \; & n=Z^{2}\;\frac{pq}{d2}\\ n=\frac{{\left(1.96\right)}^{2}0.5\left(1-0.5\right)}{{\left(0.05\right)}^{2}}\\ n=3.84*\frac{0.25}{0.0025}\\ n=384 \end{array}$$

where *n* is the required sample size, *p* is the estimated prevalence, *z* is the *z*-score at 95% CI, and *d* is the margin error of 5%.

In the study period, a total of 2154 pregnant women got ANC services. Since the number of eligible pregnant women who came to ANC service was less than 10,000, a correction formula was used to adjust the sample size.

$$\begin{array}{cc} \; & \text{nf}=\frac{\text{ni}}{1+\text{ni}/\text{nt}}\\ \text{nf}=\frac{384}{1}+\left(\frac{384}{2154}\right)=326 \end{array}$$

where nf is the final sample size, ni is the initial sample size, and nt is the total number of eligible pregnant women in Debre Tabor Comprehensive Specialized Hospital. By considering the nonresponse rate, the calculated sample size was increased by 10% and was finalized to be 359.

The number and sequence of the pregnant women who had follow-ups at Debre Tabor Comprehensive Specialized Hospital ANC unit for the study period were taken from the follow-up log book. Then the total number of attendants (2154) was divided by the sample size to determine the sampling interval, and it was 6. The first sample was selected by simple random sampling technique, and the remaining others were selected by systematic random sampling from the registered logbook.

### 2.6. Data Collection and Processing

Medical records of pregnant women who came to the hospital ANC unit were reviewed. A structured questionnaire was designed and used to collect data that contained general information about sociodemographic characteristics, medical histories, and prescribed drugs in each trimester. Experienced pharmacists who work in DTCSH gathered the data. To assure the quality of collected data, the following measures were taken: an appropriately designed data collection instrument was used, and 5% of the sample was pretested for the evaluation of the data collection instrument. The investigators ensured the data quality by checking accuracy, clarity, completeness, and consistency immediately after data collection. Ambiguities and errors that occurred were corrected after being diagnosed.

### 2.7. Data Analysis

After data collection, quantitative data was analyzed using Statistical Package for Social Science (SPSS) Version 23. Frequency, mean ± standard deviation, and percentage were used to describe the processed data in the form of tables, charts, and graphs. The association of each independent variable with the dependent variable was assessed. The variables that have an association, as explained by a *p* value of less than 0.25, were taken and further analyzed using multivariate logistic regression. Then after, a cut point of *p* value less than 0.05 was used to declare the presence of a significant association.

### 2.8. Ethical Considerations

The study was conducted after it was ethically reviewed and approved by Debre Tabor University College of Health Sciences, Department of Public Health and Department of Pharmacy. Then an official letter (with an ethical approval number of DTU 1634/2022) was taken from the university's community-based education department to the hospital. We get informed consent from the study participants verbally. The letter sent from the university was approved by the hospital administrative office after explaining the study protocol and aims of the study (with ethical approval number DTH2265/2022). For the study participants under the age of 18, guardian consent was obtained. The information that identifies the identity of the participants is not included in the questionnaire paper. No personal identification or labeling was used, and this study complied with the Declaration of Helsinki.

## 3. Results

### 3.1. Sociodemographic and Socioeconomic Information

A total of 359 patient cards of pregnant women who attended ANC follow-up at Debre Tabor Comprehensive Specialized Hospital were taken and assessed during the study period. Most of the study subjects (325/359, 90%) were married, while only 8/359 (2.2%) were single. As shown in Table 1, the largest proportion of pregnant women attending ANC follow-up (186 out of 359) were aged 20–34 years, representing 51.8%.

### 3.2. Obstetric and Medical History of Study Subjects

About 37.9% of pregnant women (136/359) were in the second trimester, while the remaining 34.8% (125/359) and 27.3% (98/359) were in the third and first trimesters, respectively. A comparable number of the study participants (133/359) were multigravida (37.0%) and secondi gravidae (130/359, 36.3%), and the remaining 96 women were primigravidae (26.7%) (Table 2).

### 3.3. Medical Disorders of Pregnant Women

As shown in Table 3, about 24 women had experienced nausea and vomiting (6.7%), followed by dyspepsia 22 (6.1%), intestinal parasite 17 (4.7%), and anemia (4.2%).

### 3.4. Prescribed Drugs and Their Pregnancy Category

A total of 602 drugs were prescribed for 350 pregnant women who visited the ANC unit of DTCSH, which gave rise to an average of 1.72 drugs per individual. Most (280/602, 46.5%) of drugs were prescribed during the second trimester, followed by 34.7% (209/602), and 18.8% (113/602) in the third and first trimesters, respectively. The most commonly prescribed medications are iron–folic acid combination (340/602, 56.5%), albendazole (48/602, 8%), mebendazole (37/602, 6.1%), omeprazole (33/602, 5.5%), and amoxicillin (32/602, 5.3%). Most (79.3%) of the drugs are from FDA Pregnancy Categories A and B, followed by 20.2% from Category C and 0.1% from Category D (Table 4).

### 3.5. Route of Administration of Drugs

**Figure 1:**
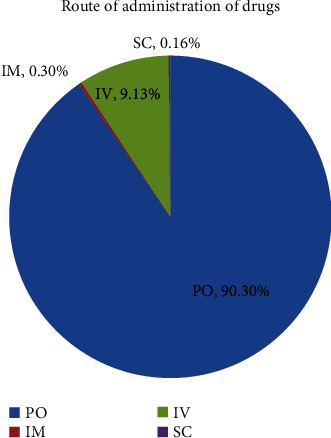
Route of administration of drugs for pregnant women attending ANC in the DTCSH, Northwest Ethiopia, June 01, 2022, to August 30, 2022.

Most prescribed drugs were taken orally (544/602, 90.3%), while the remaining were administered through IV (55/602, 9.13%), IM (18/602, 0.3%), and SC (10/602, 0.16%) routes (Figure 1).

### 3.6. World Health Organization (WHO) Prescribing Indicators

A total of 602 drugs were prescribed for 350 pregnant women, giving an average of 1.72 drugs per prescription (Table 5). About 60/359 (16.6%) pregnant women were given injections from the overall women with prescription drugs.

### 3.7. Prescription Drug Use During Pregnancy and Associated Factors

Pregnant women with the age of > 43 years (AOR = 2.23, 95% CI [1.62–3.54]) were more likely to be prescribed with drugs than young pregnant women (age 15–19 years). Similarly, pregnant women who first visited the facility in their third trimester of pregnancy were 4.32 (CI = 1.37–6.81) times more prescribed with drugs than pregnant women who had their first visit during their first trimester of pregnancy. Additionally, prescription drugs were 7.54 (2.34–14.68) times more prescribed to pregnant women having additional chronic illnesses than the women without chronic illnesses (Table 6).

## 4. Discussion

Despite limited information on the safety of drugs during pregnancy, the prevalence of drug utilization in this population is notably high [15]. In this study, the prevalence of drug utilization during pregnancy was 97.5%, which is comparable with the results of 96% in Nekemte City [16] and 93%–99% in France [17]. On the other hand, this result was greater than 87.5% of Northern Ethiopia [18], 88.4% of Bahir Dar [12], 85.1% of Harar [19], 71.3% of Addis Ababa [11], 73.2% of Cameroon [20], and 85.2% of Germany [17]. The reasons for the variations may be due to the difference in the study populations and sample size.

In the study, iron and vitamins accounted for 64% of the total prescribed drugs, with the iron–folic acid combination product being the most common, which was much higher than the result of a study done in eight districts of Ethiopia [21]. The higher utilization of the supplement in this study could be the easy and free accessibility of the health facilities and the drug in the study area and good health awareness of the urban population. Additionally, it could be due to insufficient knowledge about nutrition and a lack of budget to have a balanced diet. In contrast to this, a much higher (95%) iron/folic acid combination drug was used in Northern Ethiopia [18]. This higher drug use could be because of an unbalanced diet in northern Ethiopia associated with unproductive land, which in turn results in insufficient levels of iron and vitamins.

From the other drugs, antihelminths, antibacterials, gastrointestinal drugs, analgesics, antifungals, and antimalarials were prescribed, which took a share of about 14.1%, 9.3%, 6.3%, 4.2%, 1%, and 0.4%, respectively. This finding is in line with the studies done in Northern Ethiopia [18], Harar [19], and Oman [22]. This can be supported by the fact that diseases of poor sanitation and infectious diseases are common in developing countries. These diseases may be associated with pain and fever, which necessitate the prescription of analgesics and antipyretics. Moreover, gastrointestinal disorders, fungal infections, and malaria were also additional disorders that initiated drug prescriptions.

Based on the US-FDA classification, most of the prescribed drugs were from Categories A and B, which are safer during pregnancy. Studies from Northern Ethiopia [18] and the Netherlands [23] have reported similar prescribing patterns. On the other hand, about 0.1% of the prescribed drugs were from Category D, which are considered teratogens. This result is comparable with the findings of other studies that report utilization of less than 1% of these drugs [18]. No drug was prescribed from Category X. Drugs from Pregnancy Category D, like diclofenac, were prescribed in the third trimester of pregnancy. Unless it is a must, the prescription of such drugs should be avoided as much as possible.

Additionally, the findings of this study showed that gravidity, first visit to the facility, the presence of chronic illness, educational status, and residency had significant associations with prescription drug use in pregnant women. As the education level of the pregnant women increased, there was a lower number of prescription drugs than the less educated women. These results were ratified by similar studies conducted in different countries [24–26]. This could be possible because educated women may have good knowledge about what to do during pregnancy, which in turn reduces the number of prescribed drugs for early prevention of medical disorders. Furthermore, they may have better knowledge of the potential risks and benefits of using medications during pregnancy as compared to less educated women. While educated women in this study received fewer prescriptions, contrasting findings from Italy and Serbia suggest that increased education can lead to higher medication usage due to better access and awareness [27, 28].

Pregnant women with concomitant chronic illness were 7.5 (AOR, 95% CI [2.34–14.68]) times more prescribed with drugs than those without chronic illness. Since treatment of diseases and prevention of complications may be required for pregnant women having chronic illnesses, an increased number of prescription drugs for this group may not be surprising. This result is in concordance with the results of studies from Bahir Dar City and Cameroon [12, 20].

Furthermore, multigravida pregnant women were 1.87 (95%CI = 0.68–4.42) times more given with drugs than primigravidae pregnant women. Similar results were obtained from studies in Bahir Dar City and Cameroon [12, 20]. This could be because as the numbers of pregnancies are repeated, there may be an increased risk of maternal complications, followed by increased requirements of medications to treat these disorders. But this result is against the result of the study in Northern Ethiopia [18].

Additionally, pregnant women who first visited the facility in their third trimester get more drugs than the women visiting the facility in the first trimester (AOR = 4.32, 95% CI [1.37–6.81]). This could be because untimely resolved medical disorders proceeded to further complications, requiring additional drugs to solve these additional disorders. Moreover, it may be due to the fact that physicians feel more freedom to prescribe at the latter stages than at the early stage of pregnancy [27]. This result is in concordance with studies in Italy [24].

Our findings also showed that pregnant women residing in rural areas had more prescription drugs than women living in urban areas (AOR = 2.47, 95% CI [1.56–8.43]). This may be due to decreased awareness of rural women about how to care for themselves during pregnancy and taking an unbalanced diet, which abated the prescribers to prescribe drugs, vitamins, and iron preparations [29]. Furthermore, this could be possible because rural women may get poor sanitation, requiring additional drugs for these poor sanitation-related diseases. Similarly, pregnant women with age > 43 years are 2.23 (95% CI 1.62–3.54]) times prescribed with drugs than the women with the age of 15–19 years. The reason may be that maternal illness may increase as the age of the women increases, making them be prescribed additional drugs [30].

### 4.1. Limitation of the Study

This study is not out of limitations. Since it is a cross-sectional study, it missed past and future values of variables. Additionally, the study is conducted in a single hospital. Hence, the results are not generalizable to the country at large.

Furthermore, drug use for over-the-counter (OTC) drugs and other complementary and alternative medicines is not included in the study.

## 5. Conclusion and Recommendation

Prescription drug use was higher for pregnant women with increased age, low maternal education, chronic illness, first visit at the third trimester, and rural residency. Therefore, creating better awareness among pregnant women about what to do during their pregnancy period and initiating ANC follow-up at an early stage of pregnancy are better ways to reduce the number of prescription drugs during pregnancy. Most of the prescribed drugs were from US-FDA Categories A and B. But a small percentage of pregnant women received potentially teratogenic drugs from Category D. Prescribing teratogenic drugs should be avoided during pregnancy through increased awareness of the prescribers.

## Figures and Tables

**Table 1:** Sociodemographic and socioeconomic information of pregnant women attending ANC units at DTSH, Northwest Ethiopia, June 01, 2022, to August 30, 2022.

	**Category**	**Frequency**	**Percent**
Age	15–19	75	20.9
	20–34	186	51.8
	35–43	86	24.0
	> 43	12	3.3

Marital status	Single	8	2.2
	Married	325	90.5
	Divorced	21	5.9
	Widowed	5	1.4

Location	Urban	339	94.4
	Rural	20	5.6

Occupation	Housewife	169	47.1
	Working	190	52.9

Monthly income	< 1000	176	49.0
	1000–2000	143	39.8
	> 2000	40	11.2

Educational status	Illiterate	89	24.8
	Primary education	142	39.6
	Secondary education	42	11.7
	Higher education	86	23.9

**Table 2:** Obstetric information of pregnant women attending ANC units at DTCSH, Northwest Ethiopia, June 01, 2022, to August 30, 2022.

	**Category**	**Frequency**	**Percent**
Gravidity	Primigravidae	96	26.7
	Secondi gravidae	130	36.3
	Multigravida	133	37.0

Parity	Nullipara	123	34.3
	1–3 children	159	44.3
	> 3 children	77	21.4

Trimester	1st trimester	98	27.3
	2nd trimester	136	37.9
	3rd trimester	125	34.8

First visit to the hospital	1st trimester	221	61.6
	2nd trimester	78	21.7
	3rd trimester	60	16.7

Chronic illness	Yes	21	5.8
	No	338	94.2

Pregnancy status	Planned	306	85.2
	Unplanned	53	14.8

Number of ANC visits	1–2 times	52	14.5
	3–4 times	214	59.6
	>5 times	93	25.9

Reasons to visit the hospital	ANC	342	95.3
	Other medical cases	17	4.7

**Table 3:** Maternal disorders of pregnant women attending ANC units at DTCSH, Northwest Ethiopia, June 01, 2022, to August 30, 2022.

**Maternal disorders**	**Frequency**	**Percentage**
Iron and vitamin insufficiency	325	90.5
Anemia and vitamin deficiency	15	4.2
Malaria	2	0.6
Pneumonia	3	0.8
Vaginal bleeding	4	1.1
AFI	8	2.2
Cough and cold	6	1.8
Dyspepsia	22	6.1
UTI	7	1.9
Nausea and vomiting	24	6.7
Intestinal parasite	17	4.7
HIV/AIDS	1	0.3
Diabetes mellitus	1	0.3
Others	10	2.8

Abbreviations: AFI, acute febrile illness; HIV/AIDS, human immune virus/acquired immunodeficiency syndrome; UTI, urinary tract infection.

**Table 4:** Prescribed drugs and their FDA category for pregnant women attending ANC units at DTCSH, Northwest Ethiopia, from June 01, 2022, to August 30, 2022.

**Classes of drugs**	**Name of drug**	**Frequency of drugs in each trimester (%)**			**Total**	**Pregnancy category**
		**1st trimester**	**2nd trimester**	**3rd trimester**		
Vitamins	Iron–folic acid combination	75 (12.4%)	195 (32.4%)	70 (11.6%)	340 (56.5%)	A
	Multivitamin	0 (0%)	5 (0.8%)	23 (3.8%)	28 (4.7%)	A
	Vitamin B complex	0 (0%)	2 (0.3%)	15 (2.5%)	17 (2.8%)	A

Antibacterials	Amoxicillin	10 (1.6%)	8 (1.3%)	14 (2.3%)	32 (5.3%)	B
	Ceftriaxone	3 (0.5%)	5 (0.8%)	4 (0.7%)	12 (2.0%)	B
	Amoxicillin+clavulanic acid	1 (0.1%)	3 (0.5%)	2 (0.3%)	6 (1.0%)	B
	Ampicillin	0 (0%)	0 (0%)	3 (0.5%)	3 (0.5%)	B
	Cefalexin	0 (0%)	1 (0.1%)	2 (0.3%)	3 (0.5%)	B

Analgesics	Paracetamol	12 (2.0%)	4 (0.7%)	8 (1.3%)	24 (4.0%)	B
	Tramadol	0 (0%)	0 (0%)	1 (0.1%)	1 (0.1%)	C
	Diclofenac	0 (0%)	0 (0%)	1 (0.1%)	1 (0.1%)	D

Antihelminths	Albendazole	1 (0.1%)	12 (2.0%)	35 (5.8%)	48 (8.0%)	C
	Mebendazole	5 (0.8%)	15 (2.5%)	17 (2.8%)	37 (6.1%)	C

Antimalarial and antifungals	Coartem	0 (0%)	1 (0.1%)	1 (0.1%)	2 (0.3%)	C
	Quinine	0 (0%)	0 (0%)	1 (0.1%)	1 (0.1%)	C
	Clotrimazole	1 (0.1%)	3 (0.5%)	2 (0.3%)	6 (1.0%)	B

GI drugs	Metoclopramide	3 (0.5%)	0 (0%)	0 (0.0%)	3 (0.5%)	A
	Omeprazole	2 (0.3%)	24 (4.0%)	7 (1.2%)	33 (5.5%)	C
	Cimetidine	0 (0%)	1 (0.1%)	1 (0.1%)	2 (0.3%)	B

ART drugs	AZT/3TC/NVP	0 (0%)	0 (0.0%)	1 (0.1%)	1 (0.1%)	B

DM drugs	Insulin	0 (0%)	1 (0.1%)	0 (0.0%)	1 (0.1%)	B

Others	Anti D	0 (0%)	0 (0.0%)	1 (0.1%)	1 (0.1%)	C

Total		113 (18.8%)	280 (46.5%)	209 (34.7%)	602 (100.0%)	

**Table 5:** Prescription pattern analysis using WHO prescribing indicators for pregnant women attending ANC units of DTCSH, Northwest Ethiopia, from June 01, 2022, to August 30, 2022.

**Prescribing indicators**	**1st trimester**	**2nd trimester**	**3rd trimester**	**Total**	**Reference**
Average number of drugs per prescription	1.74 (113/65)	1.73 (280/162)	1.70 (209/123)	1.72 (602/350)	1.6–1.8
% of encounters with antibiotics	21.5% (14/65)	10.49% (17/162)	20.32% (25/123)	16% (56/350)	20%–26.8%
% of encounters with injections	4.6% (3/65)	14.81% (24/162)	25.20% (31/123)	16.6% (58/350)	13.4%–24.1%
% of drugs prescribed by generic name	100% (113/113)	100% (280/280)	100% (209/209)	100% (602/602)	100%

**Table 6:** Prescription drug use and associated factors for pregnant women attending ANC units of DTCSH, Northwest Ethiopia, from June 01, 2022, to August 30, 2022.

**Variable**	**Drugs prescribed**		**AOR (95% CI)**	**p** ** value**
	**Yes**	**No**		
Age (years)				
15–19	70	5	Ref	
20–34	183	3	1.37 (0.16–2.22)	0.001
35–43	85	1	1.81 (0.53–3.41)	0.003
> 43	12	0	2.23 (1.62–3.54)	0.001
Gravidity				
Primigravidae	92	4	Ref	
Secondi gravidae	126	4	1.43 (0.41–2.52)	0.006
Multigravida	132	1	1.87 (0.68–4.42)	0.001
Number of ANC visits				
1–2 times	47	5	Ref	
3–4 times	211	3	1.12 (0.12–5.46)	0.001
> 5 times	92	1	1.51 (0.82–7.91)	0.004
First visit to the health facility				
First trimester	214	7	Ref	
Second trimester	76	2	2.91 (1.12–6.64)	0.005
Third trimester	60	0	4.32 (1.37–6.81)	0.001
Chronic illness				
No	14	7	Ref	
Yes	336	2	7.54 (2.34–14.68)	0.001
Educational status				
Illiterate	88	1	Ref	
Primary education	139	3	0.92 (0.76–2.38)	0.001
Secondary education	41	1	0.75 (0.43–5.51)	0.001
Higher education	82	4	0.44 (0.14–2.37)	0.001
Residency				
Urban	330	9	Ref	
Rural	20	0	2.47 (1.56–8.43)	0.02

## Data Availability

Upon reasonable request, all datasets can be obtained from the corresponding author.

## Nomenclature

ANC: antenatal care
CI: confidence interval
DTCSH: Debre Tabor Comprehensive and Specialized Hospital
IM: intramuscular
IV: intravenous
PO: per os
SC: subcutaneous
SPSS: Statistical Package for Social Sciences
US-FDA: United States Food and Drug Administration
WHO: World Health Organization
